# T Cell Histiocyte Rich Large B Cell Lymphoma Presenting as Hemophagocytic Lymphohistiocytosis: An Uncommon Presentation of a Rare Disease

**DOI:** 10.1155/2017/6428461

**Published:** 2017-08-21

**Authors:** Uroosa Ibrahim, Gwenalyn Garcia, Amina Saqib, Shafinaz Hussein, Qun Dai

**Affiliations:** ^1^Department of Hematology/Oncology, Staten Island University Hospital, 475 Seaview Avenue, Staten Island, NY 10305, USA; ^2^Department of Pulmonary/Critical Care, Staten Island University Hospital, 475 Seaview Avenue, Staten Island, NY 10305, USA; ^3^Department of Pathology, Staten Island University Hospital, 475 Seaview Avenue, Staten Island, NY 10305, USA

## Abstract

T cell histiocyte rich large B cell lymphoma (THRLBCL) is a rare subtype of non-Hodgkin's lymphoma characterized by malignant B cells with reactive T lymphocytes. The pathophysiology is thought to involve cytokine-mediated evasion of T cell immune response by malignant B cells. It usually presents at an advanced stage with extranodal involvement. An extremely unusual manifestation of the disease is hemophagocytic lymphohistiocytosis (HLH) which is a hyperinflammatory disorder. We present a case of a 43-year-old male who presented with recurrent fever and recent radiologic imaging showing splenomegaly and right inguinal lymphadenopathy. On presentation, he had a fever of 105°F. Laboratory work-up was consistent with pancytopenia, elevated lactate dehydrogenase, elevated D-dimer, and a ferritin of 24,247 ng/mL. The patient was started on steroid therapy. An excisional biopsy of the right inguinal lymph node was consistent with a diagnosis of THRLBCL and the patient subsequently received six cycles of chemotherapy with R-CHOP (Rituximab, Cyclophosphamide, Doxorubicin, Vincristine, and Prednisone) after which a PET-CT scan showed no evidence of biologically active disease and ferritin was down to 822 ng/mL. We discuss the clinical manifestations and diagnostic and therapeutic considerations of this rare disease along with a review of reported cases in the literature.

## 1. Introduction

T cell histiocyte rich large B cell lymphoma (THRLBCL) is a rare subtype of diffuse large B cell lymphoma (DLBCL) characterized by malignant B cells with an infiltrate of reactive T lymphocytes. It is often an aggressive malignancy requiring prompt recognition and treatment. Hemophagocytic lymphohistiocytosis (HLH) is an uncommon hyperinflammatory disorder with an acute and potentially fatal presentation. It can be familial or acquired as a result of an underlying disorder such as an infection, malignancy, or an autoimmune phenomenon. Hematologic malignancies account for majority of the cases of secondary HLH. We describe a rare case of HLH secondary to THRLBCL presenting with persistent high fever. We discuss the unfolding of the diagnosis which can be challenging in these clinical scenarios, as well as management considerations with reference to five other cases reported to date.

## 2. Case

A 43-year-old male presented to our hospital with complaints of recurrent high fever for a few weeks. On an Urgent Care visit eight weeks prior to presentation, the patient was found to be positive for influenza. He was thereafter treated with Levaquin for persistent febrile episodes, later requiring hospitalization during which work-up revealed pancytopenia and hyperferritinemia. On computed tomography (CT) scan of the abdomen, multiple hypodense dense lesions were seen within the spleen with the largest one in the medial posterior part measuring 4.5 × 3.8 cm, and right inguinal lymphadenopathy. A bone marrow aspirate smear was adequately cellular with left shifted myeloid and erythroid maturation. A histiocyte with hemophagocytosis was seen. There was no evidence of malignancy. The patient was subsequently discharged from the facility and was scheduled for a CT-guided lymph node biopsy.

Four days later, the patient presented to our emergency department with a fever of 105°F. CBC revealed pancytopenia with a total white count of 1 × 10^9^ cells/L, hemoglobin of 7 g/dL, and a platelet count of 60 × 10^9^/L. Renal function and electrolytes were normal. Total bilirubin was 1.4 mg/dL, aspartate aminotransferase, alanine aminotransferase, and alkaline phosphatase were normal. Uric acid was 1.7 mg/dL, LDH 913 U/L, triglycerides 302 mg/dL, PT 14.7 seconds, and PTT 39 seconds. CT of the abdomen and pelvis with contrast revealed multiple enlarged abdominal lymph nodes including perigastric and pancreatic lymph nodes measuring up to 2.2 cm and retroperitoneal lymphadenopathy including a large retrocaval lymph node measuring up to 3.6 cm. There was a right inguinal conglomerate of lymph nodes measuring up to 6.6 × 4.1 cm. The spleen was enlarged with diffuse enhancement. There was no focal hepatic lesion identified. Further work-up revealed an elevated D-dimer at 7123 ng/mL and ferritin 24,247 ng/mL. Haptoglobin was less than 20 mg/dL. Rheumatologic work-up including anti-CCP antibody, SSA and B, ANA, ANCA, anti-myeloperoxidase antibody, and rheumatoid factor was negative. Viral serology including HIV and hepatitis B and C was negative. Soluble IL-2 receptor alpha was elevated at 9726 U/mL (Normal 406–100 U/mL).

**Figure 1:**
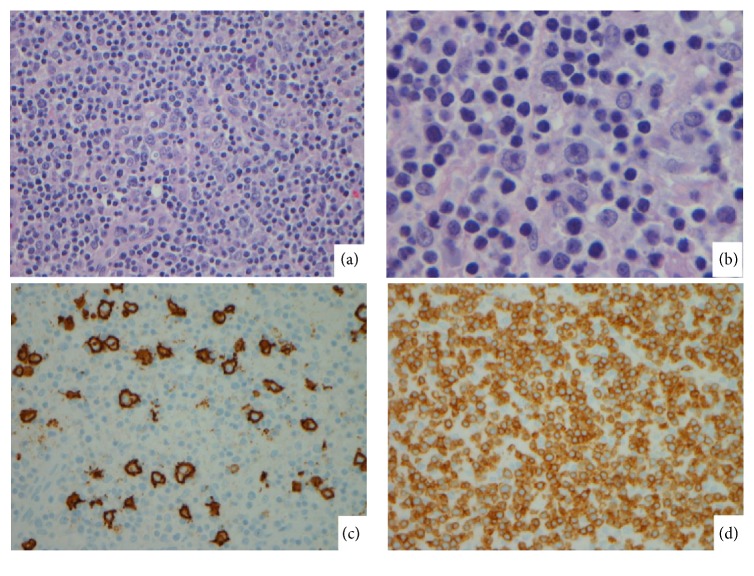
The lymph node showed effacement of normal architecture comprised predominantly of small lymphocytes and histiocytes ((a), H&E, ×200). Scattered large atypical cells with irregular nuclei and nucleoli were observed ((b), H&E, ×1000). CD20 highlights the large cells while CD3 stains numerous small T cells in the background ((c) and (d), ×400).

The differential diagnosis at this point included a lymphoproliferative disorder, hemophagocytic lymphohistiocytosis, or other inflammatory disorder. The patient had an excisional biopsy of the right inguinal lymph node that showed effacement of normal nodal architecture. The cellular elements comprised predominantly of small lymphoid cells and histiocytes with scattered large atypical cells. These atypical cells showed irregular nuclei with distinct nucleoli. Immunophenotypically, they were positive for CD20, PAX5, OCT2, BCL6, and MUM1 while they were negative for CD10, CD15, and CD30. In situ hybridization for EBV encoded RNA (EBER) was negative. The background small lymphocytes consisted mostly of T cells and histiocytes. While numerous small and some large B cells were seen in the abnormal follicles surrounding the atypical infiltrate, they were absent in the diffuse histiocyte rich areas. Small T cells positive for CD3, CD2, CD5, CD7, and CD43 were present both in the abnormal follicles and the histiocyte infiltrate. Ki 67 staining was approximately 30% with preferential staining of the larger cells [Figure 1]. The slides were sent for a second opinion and the overall findings were supportive of a diagnosis of T cell histiocyte rich large B cell lymphoma.

The patient was started on dexamethasone 20 mg given intravenously daily. He responded well and subsequently the dose of intravenous steroids was reduced and he was switched to oral Prednisone. A Positron Emission Tomography (PET) scan was performed that showed splenomegaly (15.7 cm) with multiple mass-like areas of increased uptake with a maximum SUV of 10.2. Patchy uptake was seen in the liver with a maximum SUV of 8.9 in the posterior right lobe. Multiple FDG-avid subdiaphragmatic, perigastric, peripancreatic, periportal, mesenteric, retroperitoneal, right iliac chain, and right external iliac lymph nodes were identified.

Following the diagnosis of THRLBCL, the patient was treated with R-CHOP (Rituximab, Cyclophosphamide, Doxorubicin, Vincristine, and Prednisone) given the lack of data to support use of a more aggressive regimen such as DA-R-EPOCH (dose-adjusted Rituximab, Etoposide, Prednisone, Vincristine (Oncovin), Cyclophosphamide, and Doxorubicin). A PET scan performed after three cycles of chemotherapy showed a decrease in the size of the spleen (now 13.4 cm) with conversion to non-FDG-avid status. The multiple enlarged retroperitoneal lymph nodes also decreased in size and were no longer FDG-avid. The ferritin level decreased to 3304 ng/mL. The patient completed a total of six cycles of chemotherapy without complications at the end of which his ferritin was 822 ng/mL, LDH 170 U/L, and PET-CT scan showed no evidence of biologically active disease. The patient remains in remission after six months of completion of therapy.

## 3. Discussion

T cell histiocyte rich large B cell lymphoma (THRLBCL) is a rare subtype of lymphoma accounting for 1-2% of diffuse large B cell lymphoma (DLBCL). It is histologically characterized by few scattered large malignant B cells (typically <10% of the cell population) in a background of reactive T cells and histiocytes. The pathophysiology of this disease is thought to involve cytokine-mediated evasion of the T cell immune response by the malignant B cells. It is an aggressive lymphoma, presenting at an advanced stage and with extranodal involvement in over 60% of cases. Five-year overall survival with R-CHOP is reported at 46% [1, 2].

Hemophagocytic lymphohistiocytosis (HLH) is an immune-mediated disorder characterized by fever, splenomegaly, and cytopenias. Laboratory features include hypertriglyceridemia, hypofibrinogenemia, hemophagocytosis, low or absent NK-cell activity, hyperferritinemia, and an elevated soluble CD 25. The diagnosis is made with either the presence of molecular aberrations consistent with HLH, for example, pathologic mutations in PRF1, UNC13D, or STX11, or with fulfilment of five of eight clinical criteria outlined above. The pathophysiology of HLH involves the uncontrolled activation of T cells, histiocytes, and macrophages, resulting in an overproduction of inflammatory cytokines and consequent multiorgan damage [3, 4].

Serum levels of soluble interleukin-2 receptor alpha (sIL-2R alpha) and soluble CD163 (sCD163) reflect the degree of activation and expansion of T cells and phagocytic macrophages, respectively. The IL-2 receptor complex is a trimer, consisting of alpha, beta, and gamma chains, that interact with IL-2. A soluble form of IL-2R appears in serum and plasma, concomitantly with increased cell surface expression. Serum-soluble interleukin-2 receptor (sIL-2r) level is considered an important diagnostic test and disease marker in HLH [5].

HLH may occur as a primary or familial disorder, or it may be secondary to a triggering event. Infections and malignancies are the most commonly identified triggers. Lymphoma is the most common malignancy known to trigger HLH. Primary HLH is treated with a regimen of dexamethasone and etoposide, with intrathecal methotrexate and hydrocortisone in patients with central nervous system involvement. The optimal management of malignancy-associated HLH is uncertain; HLH-directed therapy, malignancy-directed therapy, or a combination of both may be offered depending on the clinical scenario [3, 6].

Treatment of THRLBCL is on the lines of DLBCL treatment. To date, only five cases of HLH occurring in the setting of THRLBCL have been described in the English literature [7–11]. Of the prior documented cases [Table 1], two achieved complete remission (CR) with R-CHOP, with one of the patients relapsing ten months later [6, 8]. A third patient achieved CR with DA-R-EPOCH; and a fourth patient achieved CR with high-dose chemotherapy followed by autologous stem cell transplantation [9, 10]. The last patient achieved remission with salvage therapy but developed a fatal relapse several months later [11].

An intriguing fact is that THRLBCL has been shown to express cytokines such as tumor necrosis factor-*α*, interferon-*Υ*, and interleukin 6; and the same cytokines have also been implicated in the pathogenesis of HLH [2, 4, 12]. It is interesting to note that a predominantly B cell malignancy evokes a cytotoxic T cell response which is thought to be largely ineffective [13]. Genetic profiling studies have demonstrated tolerogenic immune response signatures in THRLBCL and may explain the aggressive nature of the disease. What may not be entirely coincidental is also the finding of programmed death ligand 1 (PD-L1) expression by both tumor cells and the histiocytes in the microenvironment, which may be responsible for rendering the T cells ineffective as mentioned above [14]. The question then arises is if this can be a potential target of immune therapy in the disease. Given the rarity of this condition, its acuity, and aggressiveness, chemotherapy and steroids remain the standard treatment. The role of immune modulation, apart from glucocorticoids, is yet to be explored.

Treatment options for refractory or recurrent disease include alemtuzumab, a monoclonal antibody to the CD52 protein expressed on the surface of mature T cells and possibly NK cells. This acts as a bridge towards hematopoietic stem cell transplant. Patients with hematologic malignancies may have recurrent episodes of HLH given that the malignancy persists as a trigger. These patients must be referred for transplant which can be allogeneic or autologous depending on the primary disease. Avenues of ongoing research include an anti-interferon-gamma monoclonal antibody and the study of pegaspargase together with liposomal Doxorubicin, etoposide, and high-dose methylprednisolone (L-DEP) as an initial treatment for Epstein Barr virus-induced hemophagocytic lymphohistiocytosis [15].

## 4. Conclusion

HLH is a potentially fatal condition and when it is secondary to an aggressive malignancy, prompt diagnosis and initiation of treatment can be life-saving for the patient. While awaiting pathology results, treating the HLH is important for symptom control; however, treating the primary disease remains a priority in the long-term management of patients.

## Figures and Tables

**Table 1:** Reported cases of hemophagocytic lymphohistiocytosis secondary to T cell histiocyte rich large B cell lymphoma.

Serial #	Age/sex [ref]	Presentation	Site of involvement	Immunophenotype	Treatment	Outcome
1	20/M [7]	Jaundice, malaise, abdominal pain, fever	LN, liver	CD3+ CD5+ CD7+ CD45+ T cell infiltrate Scattered large CD20+ PAX5+ CD15− CD30− Alk-1− B-cells	R-CHOP × 6 IT-MTX	Alive

2	52/M [8]	Fever, DOE, weight loss	LN	Scattered large CD20+ cells CD3+ T cells CD30− CD15− EBV−	R-CHOP × 8, IT-MTX, cytarabine, MP	Recurrence at 10 m, salvage therapy

3	30/M [9]	Fever, jaundice, weight loss, ARF	LN, lung	Large CD20+ CD15− CD30− B cells CD3+ CD5+ CD7+ CD8+ TIA-1+ T cells CD68+ histiocytes	DA-R-EPOCH	Alive

4	30/F [10]	Pruritus, night sweats, fever, weight loss	LN, liver	CD79a+ Mib-1+ large cells	MOPP-ABV then high dose MTX, vincristine and etoposide, then AHSCT	Alive at 24 m

5	34/M [11]	Fever, abdominal pain, jaundice	BM	ND	ND	DOD

6	43/M [current case]	Fever	LN	Large atypical cell CD20+, PAX5+, BCL-6+, MUM1+, EMA (weak), Kappa (weak) CD3+, CD2+, CD5+, CD7+, CD43+ T cells	R-CHOP × 6	Alive

AHSCT: autologous hematopoietic stem cell transplant; BM: bone marrow; DA-R-EPOCH: dose adjusted Rituximab, Etoposide, Prednisone, Vincristine (Oncovin), Cyclophosphamide, and Doxorubicin; DOE: dyspnea on exertion; DOD: died of disease; IT-MTX: intrathecal methotrexate; LN: lymph node; M: months; MOPP-ABV: mechlorethamine, vincristine, procarbazine, prednisone/doxorubicin bleomycin, and vincristine; MP: methylprednisolone; ND: not described; R-CHOP: Rituximab, Cyclophosphamide, Doxorubicin, Vincristine, and Prednisone.
